# Potential phytoestrogen alternatives exert cardio-protective mechanisms *via* estrogen receptors

**DOI:** 10.1051/bmdcn/2017070204

**Published:** 2017-06-14

**Authors:** Marthandam Asokan Shibu, Wei-Wen Kuo, Chia-Hua Kuo, Cecilia-Hsuan Day, Chia-Yao Shen, Li-Chin Chung, Chao-Hung Lai, Lung-Fa Pan, V. Vijaya Padma, Chih-Yang Huang

**Affiliations:** 1 Graduate Institute of Basic Medical Science, China Medical University Taichung 404 Taiwan; 2 Department of Biological Science and Technology, China Medical University Taichung 404 Taiwan; 3 Laboratory of Exercise Biochemistry, Department of Sports Sciences, University of Taipei Taipei 100 Taiwan; 4 Department of Nursing, Meiho University Pingtung 912Taiwan; 5 Department of Hospital and Health Care Administration, Chia Nan University of Pharmacy & Science Tainan 717 Taiwan; 6 Division of Cardiology, Department of Internal Medicine, Armed-Force, Taichung General Hospital Taichung 411 Taiwan; 7 Department of Biotechnology, Bharathiyar University Coimbatore Tamil Nadu 641046 India; 8 School of Chinese Medicine, China Medical University Taichung 404 Taiwan; 9 Department of Health and Nutrition Biotechnology, Asia University Taichung 413 Taiwan

**Keywords:** 17 Beta-estradiol, Estrogen receptor-alpha, Estrogen receptor-beta, Phytoestrogens, Survival signalling, Apoptosis

## Abstract

The 17 beta-estradiol (E2) is a sex hormone that is most abundant and most active estrogen in premenopausal women. The importance of E2 in providing cardioprotection and reducing the occurrence of heart disease in women of reproductive age has been well recognized. There are three subtype of estrogen receptors (ERs), including ERα, ERβ and GPR30 have been identified and accumulating evidence reveal their roles on E2-mediated genomic and nongenomic pathway in cardiomyocytes against various cardiac insults. In this review, we focus on the estrogen and ERs mediated signaling pathways in cardiomyocytes that determines cardio-protection against various stresses and further discuss the clinical implication of ERs and phytoestrogens. Further we provide some insights on phytoeostrogens which may play as alternatives in estrogen replacement therapies.

## 1. Introduction

Cardiovascular disease is the leading cause of death worldwide. Millions of patients succumb to the consequences of myocardial ischemia, heart failure, and arrhythmias. Heart disease usually develops as a result of the deterioration of function of the myocardium, which leads to heart failure. The incidence of heart disease is low in premenopausal women but increases substantially after menopausal, suggesting that sex steroid hormones protect the female heart [1]. Cardiovascular cells express estrogen receptors (ERs) that are important targets for endogenous estrogen. Estrogen-ER complexes serve as transcription factors that promote gene expression which fortifies cardiac health [2]. Accumulating evidence from *in vivo* and *in vitro* studies suggests that 17 beta-estradiol (E2); the most abundant and active estrogen in premenopausal women; contribute to cardioprotection by preventing cardiomyocyte apoptosis and by alleviating left ventricular hypertrophy and cardiac fibrosis in women. Although estrogen replacement therapies on postmenopausal women and ovariectomized animal models are reported to suppress cardiac cell survival they promote other cardio vascular complications [3, 4].

**Fig. 1:**
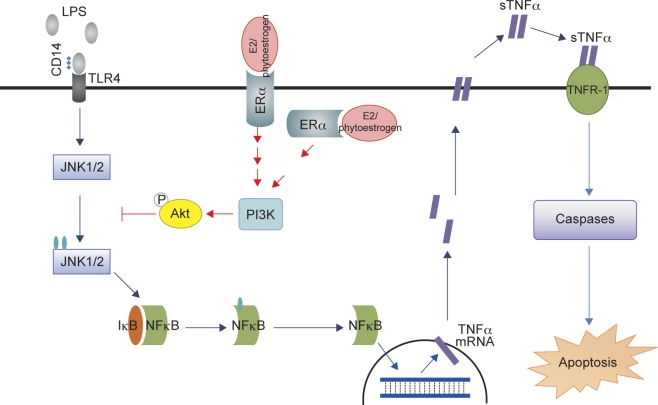
Anti-apoptotic mechanism of phytoestrogen against extrinsic apoptosis: Similar to E2, phytoestrogens elevates the activity of PI3K-Akt to inhibit the JNK1/2 mediated activation and nuclear translocation of NFkB and thereby alleviates the LPS-induced cardiomyocyte apoptosis.

**Fig. 2:**
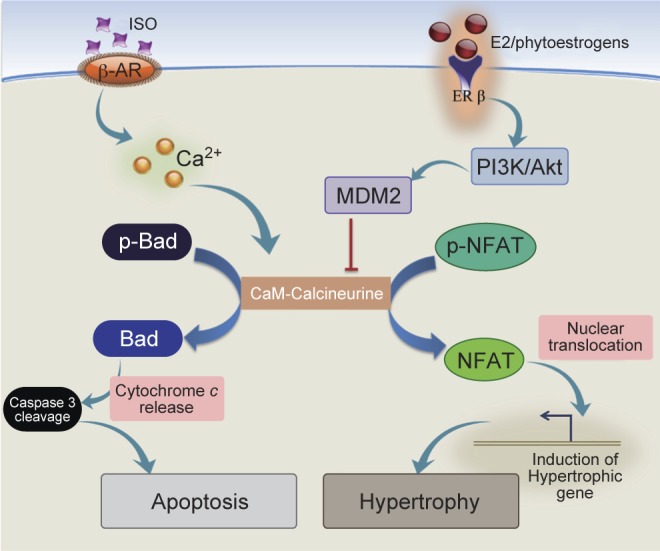
Anti-apoptotic mechanism of phytoestrogens against mitochondria associated apoptosis. Phytoestrogens potentially enhance PI3K-Akt activity and alleviates calcium accumulation in the cardiomyocytes by inhibiting the calcineurin mediated dephosphorylation of Bad to attenuate ISO induced apoptosis.

**Fig. 3:**
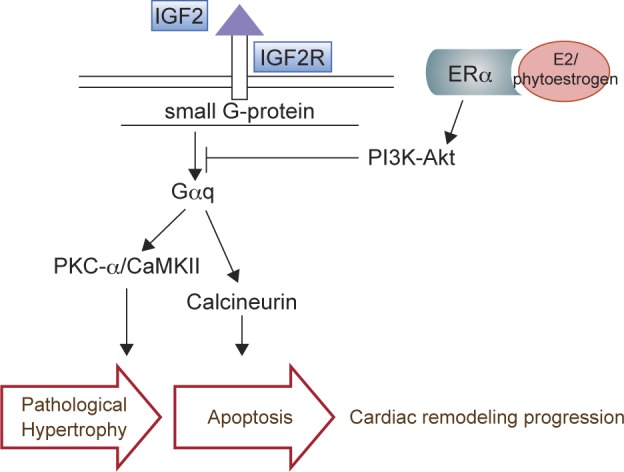
Phytoestrogens ameliorate IGF2R mediated cardiomyocyte apoptosis. Alike E2phytoestrogens may act against IGF2R-induced hypertrophy and cardiomyocyte death by inhibiting the Gαq mediated activation of cakineurin.

Therefore, further investigations are needed to fully understand the complex effects of estrogen and ERs on cardiomyocyte biology prior to its clinical application for cardiomyopathy treatment. This article will outline the basic molecular understanding of estrogen and ERs and review the current state of knowledge of estrogen signaling in cardiomyocyte protection. Further highlight the current knowledge on potential analogs such as the phytoestrogens that can be used to enhance cardiac health.

## 2. Structural characteristics of ERs and the events in E2/ER signaling

The most well-known physiological actions of E2 are mediated by two ER subtypes, ER-alpha (ERα) and ER-beta (ERβ), belonging to the member of the nuclear receptor superfamily and possess similar structural characteristics. The ERα and ERβ are transcribed from different genes and display distinct expression patterns as well as different ligand specificities [5]. These ERs constitutes five distinct domains that include A/B, C, D, E and F domains [6]. The A/B domain in ERα and ERβ share less than 20% amino acid homology and thereby contribute ER subtype-specificity on target genes. The activation function 1 element of the A/B domain provides the ability of ligand-independent ER activation [7].

The central C-domain is critical for specific DNA binding and dimerization of ERs and D-domain is important for ER nuclear translocation and is the hinge domain between the DNA-binding domain (DBD) and the E-domain which harbors a hormone-dependent activation function 2 (AF-2) element [8, 9]. The functions of the F-domain are not unclear.

Recently, the estrogen receptor GPR30 was identified as an orphan G-protein coupled receptor and estrogen is its endogenous ligand. As a transmembrane ER, GPR30 activation may mediate E2 rapid cell signaling [10]. The exact effects and molecular mechanism of GPR30 in cardiomyocytes has not been fully evaluated yet.

Estrogens bind to ERs to form the nuclear estrogen-ER complex which then binds to estrogen response element sequences (ERE) in the regulatory regions of the respective estrogen responsive genes, resulting in the appropriate expression. ERs modulate gene expressions by either binding to the ERE-mediated signaling or even by interacting directly or indirectly with transcription factors; this phenomenon is known as genomic ER signaling. Alternatively in non-genomic ER signaling, estrogen binds to ERs *via* cytoplasmic signal transduction proteins, such as mitogen activated protein kinase (MAPKs), Stats (signal transducers and activators of transcription), and Src family tyrosine kinases, or through membrane-associated estrogen-binding receptors, resulting in cellular responses [11]. ER bind to phosphoinositide-3 kinase (PI3K) to increase PI3K activity and thereby activates Akt to enhance pro-survival signaling in cardiomyocytes [12]. Growth factors such as insulin-like growth factor 1 (IGF1) and epidermal growth factor (EGF) also interact with respective receptor tyrosine kinases and induce mitogen-activated protein kinase signaling, which in turn activates ER [13]. The third estrogen receptor, known as GPR30 (G-protein coupled estrogen receptor) was recently identified as the membrane-localized estrogen receptor and is located primarily on the plasma, endoplasmic reticulum and nuclear membrane [14].

## 3. Phytoestrogens: as a replacement to estrogen?

An increasing number of reports on hormone replacement therapy show that there is growing scientific interest focused on alternative therapies for postmenopausal women with a predisposition to CVD. Phytoestrogens are interesting candidates in this regard since they are structurally similar to estrogens [15]. They act as both estrogenic agonists and antagonists. Numerous herbs and medicinal plants are known to contain components similar t estrogen. Danshen is used widely in China for the treatment of cardiovascular disorders, including coronary heart disease. Danshen possesses lipid-soluble biologically active components with a structure similar to 17β-estrodiol (E2) [16]. Salvianolic acid B is an effecient phytoestrogen purified from danshen that show cardio protective effects by suppressing the apoptotic effect of treatment with high glucose combined with hypoxia in embryonic stem cell derived cardiomyocytes [17]. Resveratrol is a phytoestrogen naturally found in grapes and is a major constituent of wine thought to exert both cardioprotective and chemopreventive activities [18]. Recent studies show that this bioflavonoid binds to and activates gene transcription *via* ERα and ERβ. A recent report shows that Resveratrol enhanced FOXO3 phosphorylation *via* synergetic activation of SIRT1 and PI3K/Akt signaling to improve the effects of exercise in elderly rat hearts [19]. Epigal-locatechin-3-gallate (EGCG) is another phytoestrogen, abundant in green tea, which is known to inhibit cardiac myocyte apoptosis and oxidative stress in pressure overload induced cardiac hypertrophy [20]. Also, EGCG prevents cardiomyocyte apoptosis from oxidative stress *in vitro* [21]. Substantial evidence suggests that EGCG acts as an antioxidant by attenuating lipid peroxidation caused by various forms of ROS [22], thereby reducing the expression of the endogenous nitric oxide synthase inhibitor asymmetric dimethyl arginine [23] as well as reducing the expression of cytokine-induced vascular adhesion molecule-1 [24]. EGCG also prevents the oxidized low-density lipoprotein-induced LOX-1-mediated biological events that are closely linked to endothelial dysfunction.

## 4. Phytoestrogen potentially modulate E2/ERs signaling to establish protection against LPS-induced myocardial cell death.

Lipopolysaccharides (LPSs), the outer-membrane component of Gram-negative bacteria, elevate inflammation and apoptosis by interacting with toll-like receptor-4 (TLR-4) resulting in sepsis-induced heart failure and [25, 26]. The LPS-induced myocardial apoptosis is mediated by JNK1/2, which promotes the activation of NFkB, leading to the increase of pro-apoptotic proteins such as TNFα (Tumor necrosis factor), active caspases-8, t-Bid, Bax, released cytochrome c, active caspase-9 and active caspase-3 [27]. The elevated PI3K-Akt activity mediated by E2 and ERα, contributing to the inhibition of nuclear translocation of NFkB and therefore diminishes the LPS-induced cardiomyocyte apoptosis (Fig. 1) [28]. This may suggest how menopausal women with sepsis have lower mortality and heart failure incidence.

Notoginsenoside R1 isolated from *Panax notoginseng,* is a major phytoestrogen effective against inflammation and apoptosis. Notoginsenoside R1 is known to regulate LPS induced NF-kB activation and related inflammation signaling involving TNF-a, IL-1b and IL-6. The mechanism by which Notoginsenoside R1 ameliorates LPS induced cardiac dysfunction involves activation of ERα and t PI3K/Akt survival signaling pathway [29]. Resveratrol is yet another phytoestrogen that suppresses proinflammatory cytokine production and prove to be effective against LPS endotoxin myocardial injury in mice [30, 31]. Apigenin, a flavone found in many fruits, vegetables and nuts are also known to reduce inflammation associated cardiac injury. Apigenin is known to down-regulate LPS induced inflammation associated TNF-α and IL-6 and IL-1β levels in heart and apoptosis associated cleaved caspase-3, cleaved caspase-9 and Bax levels in rat hearts [32].

## 5. Phytoestrogens modulate E2/ERs signaling and protects against apoptosis induced by hypertrophic agents

Cardiac hypertrophy is one of the most frequent causes of heart failure and it could results Cardiac insults like hypertension or by various hypertrophic agents [33–36]. The early compensative hypertrophy often progress to become pathological, resulting in cardiomyocyte apoptosis and leading to eventual deterioration of cardiac function.

Calcium-sensitive phosphatease, calcineurin play a major role in cardiac hypertrophy. The calcineurin activity is increased by a variety of isoproterenol (Iso) [36]. Calcineurin activation promotes the NFAT3 nuclear translocation and activates MEF2 (myocyte-enhancing factor 2), resulting in the up-regulation of hypertrophic genes [37].

The results from various animal studies indicate that estrogen may defend against the development of cardiac hypertrophy.

We recently showed that E2 and ERβ alleviate isoproterenol-induced cellular calcium accumulation in cardiomyocytes by activating phospholamban and PI3K-Akt-MDM2 signaling cascades [38, 39] which increase the Q5 protein degradation of calcineurin. Therefore, E2/ ERβ inhibit isoproterenol-induced myocardial cell hypertrophy and apoptosis (Figure 2). On the other hand, ERa seems to have distinctive protective mechanism against Iso-induced hypertrophy and apoptosis in myocardial cells. Our lab has recently indentified that E2 facilitate the increase of interaction between ERa and Src results in the activation of IGFIR-PI3K-Akt and EGFR-MMP2/9-MEK1/2-ERK1/2 signaling pathways thereby reduces the calcineurin induced pro-apoptotic protein levels and protects cardiomyocytes from Iso-induced apoptosis (results from our unpublished data). E2 activates Src and heparin-bound epidermal growth factor (EGF) and thereby activates the EGF receptor with subsequent acute activation of PI3K and ERK activation [40].

Treatment with Genistein on H9c2 cardiomyoblasts suppresses the Iso induced apoptosis by reducing the mitochondrial pro-apoptotic proteins including Bad, caspase-8, caspase-9, and caspase-3 and by enhancing p-Akt, p-Bad, and p-Erk1/2 [41]. Phytoestrogen genistein has been shown to attenuate the development of pulmonary hypertension through Erβ [42]. Genistein has been proven to prevent phenylephrine induced cardiac muscle hypertrophy by inhibiting ERK1/2 and also potentially attenuate pressure overload-induced cardiac hypertrophy and fibrosis [43, 44].

## 6. Phytoestrogens mimic E2/ERs mediate cardio-protection against oxidative stress and ischemic-reperfusion injury

Ischemic heart disease (IHD) attributes to myocardial infarction or traumatic injury and it is one of the major cause of death and hospitalization in many countries [45]. The reduced blood supply induces a hypoxic situation of the heart muscle, which usually stimulates cytokines production, such as IL-6, leading to inflammation in myocardium and cardiomyocytes apoptosis [46, 47]. Ischemia-reperfusion injury is the damage caused by reestablishment of blood flow and re-oxygenation to the infracted tissue. Premenopausal women have lower incidence of IHD than men of same age probably due to the role of E2 and various epidemiological studies also indicate the same [48]. Estrogen replacement therapy reduces the infarct size and reduces of apoptosis in the peri-infarct zone of the left ventricle in coronary artery ligation animal model, suggesting that estrogen can effectively protect hearts against myocardial ischemia reperfusion injury [49].

Hypoxia-induced BNIP3 plays an important role in the development of hypoxia-induced cardiac hypertrophy and cardiomyocyte death [50]. We have recently found that ERα may bind to the regulatory region of BNIP3 gene, which is probably located on AP-1 or NFkB binding site within the promoter, leading to the suppression of BNIP3 transcriptional level. Meanwhile, the binding of ERα and BNIP3 proteins seems to facilitate proteasomes-mediated degradation of BNIP3. These two distinct ERα meditated effects contribute to the reduction in the BNIP3-induced cardiomyocyte apoptosis (results from our unpublished data). On the other hand, the elevated levels of oxidative stress and reactive oxygen species (ROS) during the reperfusion stage of ischemia-reperfusion injury also cause cardiomyocyte death [51, 52]. E2, either by activating ERα or ERβ, results in the activation of PI3K with subsequent inhibition of ischemia-reperfusion injury induced ROS and attenuation of apoptosis in the ischemic area [53–56].

Dietary supplementation of phytoestrogens protect heart from ischemia, followed by normothermic reperfusion in rats by maintaining the ER mediated NO release, mitochondrial structure and function and by attenuating myocardial Ca^2+^accumulation [57]. In rats EGCG have been shown to protect myocardial reperfusion injury by specific inhibition of the IKK/NF-kB and AP-1 pathway [58]. Intravenous administration of genistein effectively attenuates cell death in the myocardial necrosis and MPO activity in the infracted tissue, and improves myocardial contractile function by limiting the inflammatory response after ischaemia-reperfusion injury in animal models [59, 60]. Experimental and clinical investigation show that they phytoestrogen danshen protects hearts against hypertrophy and ischemia-reperfusion injury [61].

## 7. E2/ERs and the analogous phytoestrogens mediate cardio-protection against IGF2R death signal

Insulin-like growth factors receptor II (IGF2R) or the cation-independent mannose-6-phosphate receptor, is a protein that in humans was initially thought to be responsible for quenching IGF2 ligand [62]. Activation of IGF2R in cardiomyocytes is induced by hypertension, AngII and Inomycin. Suppression of IGF2R leads to cardiomyocytes apoptosis and leads to the MMP2/9 activation and myocardial ECM remodeling [63-65]. Our recent investigations show that the activation of PI3K-Akt pathway by E2 markedly attenuated IGF2R-induced apoptosis and hypertrophy in cardiomyocytes. These data indicate that E2 has protective effects against IGF2R-induced hypertrophy and cardiomyocyte death (Fig. 3). More research is necessary to characterize whether this protection is ER-dependent and to reveal the precise mechanisms responsible for this cardioprotection.

Our previous findings show that Danshen acts through ER to activate Akt and attenuate activation of IGF-II receptor signaling and inhibit apoptosis [66].

## 8. Conclusions and future perspective

ERα, ERβ confer cardioprotective effects against various stresses by preventing myocardial cell apoptosis and cardiac function. Our laboratory is investigating the possible regulation of autophagy by estrogen and ER-mediated cardioprotection. This is critical to understand the complex nature of cellular mechanisms of estrogen and ERs in various heart diseases, such as ischemic injury, cardiac hypertrophy, cardiac remodeling, and heart failure. Further we evaluate various phytoestrogens in promoting cardiac cell survival and function which would enable in the process of establishing alternatives for estrogen replacement treatments.
